# Protein Folding Activity of the Ribosome is involved in Yeast Prion Propagation

**DOI:** 10.1038/srep32117

**Published:** 2016-09-16

**Authors:** Marc Blondel, Flavie Soubigou, Justine Evrard, Phu hai Nguyen, Naushaba Hasin, Stéphane Chédin, Reynald Gillet, Marie-Astrid Contesse, Gaëlle Friocourt, Guillaume Stahl, Gary W. Jones, Cécile Voisset

**Affiliations:** 1 Inserm UMR 1078, Université de Bretagne Occidentale, Faculté de Médecine et des Sciences de la Santé; Etablissement Français du Sang (EFS) Bretagne; CHRU Brest, Hôpital Morvan, Laboratoire de Génétique Moléculaire, Brest, France; 2 Yeast Genetics Laboratory, Department of Biology, Maynooth University, Maynooth, County Kildare, Ireland; 3 Institute for Integrative Biology of the Cell (I2BC), UMR 9198, CEA, CNRS, Université Paris-Sud, CEA/Saclay, SBIGeM, Gif-sur-Yvette, France; 4 Université de Rennes 1, CNRS UMR 6290 IGDR, Translation and Folding Team, Rennes, France; 5 Laboratoire de Biologie Moléculaire Eucaryotes, CNRS, Université de Toulouse, Toulouse, France

## Abstract

6AP and GA are potent inhibitors of yeast and mammalian prions and also specific inhibitors of PFAR, the protein-folding activity borne by domain V of the large rRNA of the large subunit of the ribosome. We therefore explored the link between PFAR and yeast prion [*PSI*^+^] using both PFAR-enriched mutants and site-directed methylation. We demonstrate that PFAR is involved in propagation and *de novo* formation of [*PSI*^+^]. PFAR and the yeast heat-shock protein Hsp104 partially compensate each other for [*PSI*^+^] propagation. Our data also provide insight into new functions for the ribosome in basal thermotolerance and heat-shocked protein refolding. PFAR is thus an evolutionarily conserved cell component implicated in the prion life cycle, and we propose that it could be a potential therapeutic target for human protein misfolding diseases.

The infectious proteins concept was first established for the prion protein PrP in mammals with transmissible spongiform encephalopathy. In its PrP^Sc^ prion conformation, PrP accumulates as self-propagating amyloid fibers without prion-specific nucleic acid. Proteins behaving like prions have also been identified in the budding yeast *S. cerevisiae,* although it has no PrP homolog. The best studied yeast prions are [*PSI*^+^] and [URE3]: heritable amyloids of translation release factor Sup35p and nitrogen catabolism Ure2p regulator, respectively^1^,^2^.

Hsp104p is a cell factor known to be essential to prion propagation in yeast, in collaboration with heat-shock protein chaperones such as Hsp70p and Hsp40p, characterized as prion propagation modulators^3^. Hsp104p is a hexameric, ring-shaped ATPase of the AAA+ family with disaggregase, unfoldase and translocase activities^4^. In yeast, it has a prime role in disassembling and remodeling the aggregated proteome in collaboration with Hsp70 and Hsp40, thereby providing thermotolerance^5^. Hsp104p also exhibits operational plasticity adaptable to the needs of the yeast proteome, including severing prion fibers by threading Sup35p through its hexameric pore^6^. In the current model, it thus acts as a “molecular sonicator” to transform highly aggregated dead-end fibers into low-molecular-weight oligomers that can seed new rounds of polymerization and enable efficient transmission from mother to daughter cells^1^,^7^. Thus, when Hsp104p is inhibited (e.g., by guanidine hydrochloride GdnHCl) or when *HSP104* is deleted, cells are cured of [*PSI*^+^] prion, as fibers form and grow but are not severed to make new seeds. Hence, the number of fibers and seeds per cell decreases as cells divide, leading to prion-free cells^1^. In addition, Hsp104p overexpression cures [*PSI*^+^] prion, likely by complete prion fiber disaggregation to a soluble form; but the mechanism remains to be fully deciphered^7^,^8^,^9^. Metazoans lack Hsp104p orthologs; none of their AAA+ protein superfamily ATPases displays amyloid disaggregation comparable to Hsp104p^5^.

In certain lab strains, [*PSI*^+^] prion-containing cells are easily differentiated from [*psi*^−^] counterparts by color. In [*psi*^−^] cells, Sup35p is soluble and causes the ribosome to stop translation at stop codons, including the premature stop codon in the *ADE1* gene *ade1-14* allele, leading to truncated non-functional Ade1 protein. Without Ade1p, colonies become red on YPD-rich medium as cells accumulate an intermediate metabolite of the adenine biosynthesis pathway^1^. When most Sup35p is sequestrated as amyloid, it fails in its role of translation termination, and the ribosome has an increased the tendancy to read through stop codons, including *ade1-14* premature stop codon. Consequently, *ade1-14* [*PSI*^+^] cells produce functional Ade1p enzyme and form white or pink colonies on YPD-rich medium. This white/red colorimetry was used for an antiprion drug-screening assay^10^. Using successive screenings for drugs able to cure the unrelated [*PSI*^*+*^] and [URE3] yeast prions as well as PrP^Sc^ mammalian prion, we showed some mechanisms controlling prion propagation to be conserved from yeast to humans^10^,^11^. These screenings identified several new antiprion compounds: 6-aminophenanthridine (6AP), guanabenz (GA, already used in hypertension) and imiquimod (IQ, a TLR7 agonist), all active against yeast and mammalian prions *in vitro* and *in vivo*^10^,^12^,^13^. 6AP and GA were independently shown to interact with specific domain V nucleotides of the large rRNA of the large 60S ribosome subunit^14^. Domain V of the large rRNA (23S in *E. coli*, 25S in *S. cerevisiae,* 28S in metazoa) has 2 enzymatic activities, i) a peptidyl transferase activity and ii) a poorly characterized protein folding activity, PFAR: Protein Folding Activity of the Ribosome (in ref. 15; for reviews see^16^,^17^,^18^). PFAR was characterized by *in vitro* refolding experiments, showing that domain V of the large rRNA from bacteria, yeast or Drosophila assist protein folding of various denatured protein substrates^14^,^17^,^19^. PFAR was mainly studied *in vitro* and its biological role remains unclear. 6AP and GA are the first specific competitive inhibitors of PFAR not affecting ribosome peptidyl transferase activity^11^,^20^.

6AP, GA and IQ antiprion compounds display anti-PFAR activity, suggesting that PFAR may play an important role in prion generation and propagation. Investigating links between PFAR and yeast prion [*PSI*^+^], we found PFAR to be involved in the propagation and *de novo* formation of [*PSI*^+^] *in vivo.* As these properties are reminiscent of Hsp104p, we explored the interplay between PFAR and Hsp104p and showed that PFAR and Hsp104p partially compensate each other for [*PSI*^+^] propagation. This is clear evidence of PFAR’s important role with fundamental implications for protein folding and perhaps for human protein misfolding diseases.

## Results

### [*PSI* ^+^] propagation and *de novo* appearance are affected in PFAR-enriched cells

**Figure 1:**
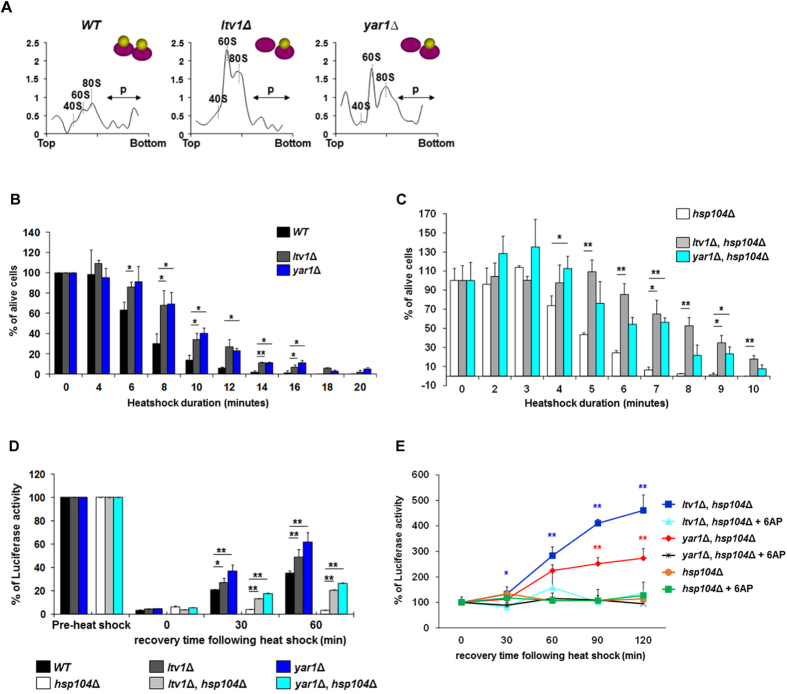
l*tv1*Δ and *yar1*Δ strains display PFAR enrichment. **(A)** Polysome profiles of *WT, ltv1*Δ *and yar1*Δ strains showing that *ltv1*Δ and *yar1*Δ mutant strains display elevated levels of free 60S subunits, as well as an impoverishment in 40S subunits compared to *WT* strain. 60S:40S ratios were 2.28 for the *WT* strain, 4.03 and 5.09 for *ltv1*Δ *and yar1*Δ strains, respectively. The y axis shows arbitrary units. **(B,C)** Basal thermotolerance of PFAR-enriched strains. *WT*, *ltv1*Δ and *yar1*Δ [*psi*^−^] cells **(B)** or *hsp104*Δ*, ltv1*Δ*/hsp104*Δ and *yar1*Δ*/hsp104*Δ [*psi*^−^] cells **(C)** were heat-shocked at 47 °C and aliquots harvested at specified incubation times and spread on YPD medium. Survival is the percentage of cells alive after heat shock compared to cells without heat shock. **(D)** Protein refolding in PFAR-enriched strains. *WT*, *ltv1*Δ*, yar1*Δ*, hsp104*Δ*, ltv1*Δ*/hsp104*Δ and *yar1*Δ*/hsp104*Δ [*psi*^−^] cells were heat-shocked at 45 °C for 60 min. Protein synthesis was inhibited by cycloheximide and aliquots were harvested after specified post-heat-shock incubation periods, and luciferase activity was measured. **(E)** *hsp104*Δ*, ltv1*Δ*/hsp104*Δ and *yar1*Δ*/hsp104*Δ [*psi*^−^] cells were pretreated by 200 μM 6AP for 2 hrs and heat-shocked at 45 °C in continuous presence of 6AP for 1 hr as described above. Experiment were repeated 3 times. A representative assay including 3 technical repeats is shown with error bars. Bar height in panels (B–D) represents the mean; *P < 0.05 or **P < 0.001 on *t*-test, relative to *WT* strain (panels B,D), *hsp104*Δ strain (panels C,D) or 6AP-treated cells (panel E).

To determine whether PFAR is linked with prionization mechanisms *in vivo* in yeast, we first investigated the effect of PFAR enrichment on [*PSI*^+^] propagation. Several studies have shown that the protein folding activity of the ribosome is silenced when translation occurs^15^,^17^,^21^ and Mondal *et al*. recently demonstrated that a tRNA positioned at the P-site inhibits the ribosome’s chaperoning function^22^. We thus created yeast mutants enriched in free 60S subunits. The rational of our approach is that 60S free subunits are more prone to fulfill protein folding function than actively translating 80S ribosomes because they are not engaged in translation^23^. The folding process has indeed been shown to start with the full-length polypeptide chain splitting a non-translating ribosome into subunits^23^. Domain V nucleotides of the large rRNA that harbor PFAR are thereby easily accessible at the surface of the 60S subunit^16^,^18^. The large 60S subunit of the ribosome is actually able to fold proteins more efficiently than the whole ribosome *in vitro,* the small 40S subunit attenuating PFAR by partly masking domain V nucleotides that harbor PFAR^21^,^23^,^24^. This post-translational protein folding event is actually assumed to be a cooperative process to permit ribosome recycling^17^. We thus created 60S-enriched yeast mutants by deleting *LTV1* and *YAR1* genes in a [*PSI*^+^] strain: Ltv1p is involved in small 40S subunit transport from nucleus to cytoplasm, while Yar1p prevents aggregation of Rps3, a protein essential for 40S subunit maturation prior to translocation from nucleus to cytoplasm^25^,^26^,^27^. Compared to *WT* strain, *ltv1*Δ and *yar1*Δ mutant strains actually showed high rates of free 60S subunits and relative impoverishment in 40S subunits (Figs 1A and S1A).

To prove functional PFAR enrichment, we assessed whether enrichment in 60S increased protein-folding capacity in *ltv1*Δ and *yar1*Δ strains and first explored basal thermotolerance (non-induced thermotolerance). *WT*, *ltv1*Δ and *yar1*Δ [*psi*^−^] strains were subjected to high temperature and surviving cells were scored at various times in both *HSP104* and *hsp104*Δ backgrounds. *ltv1*Δ and *yar1*Δ mutants proved more thermoresistant than *WT* strain (Fig. 1B), and also when combined with *hsp104*Δ versus *hsp104*Δ alone (Fig. 1C). Thus, PFAR enrichment conferred basal Hsp104-independent thermoresistance to *ltv1*Δ and *yar1*Δ strains. We next evaluated the folding capacity of *ltv1*Δ and *yar1*Δ mutant strains by monitoring *in vivo* luciferase refolding following denaturation by heat shock: it proved more efficient in *ltv1*Δ and *yar1*Δ mutants than in *WT* strain (Fig. 1D) without affecting cell survival (Figure S1B). Remarkably, *ltv1*Δ and *yar1*Δ mutants combined with *hsp104*Δ showed better luciferase refolding than *hsp104*Δ alone (Fig. 1D) together with a better survival rate (Figure S1B), which is consistent with data shown in Fig. 1B,C. This enhanced folding capacity was abolished in presence of 6AP (Fig. 1E): i.e., PFAR clearly influences the cellular response to heat shock, and is thus a protein chaperone genuinely involved in cellular refolding. Thus *ltv1*Δ and *yar1*Δ mutants displayed patently enhanced PFAR activity and are thus well suited to investigate PFAR involvement in [*PSI*^+^] prion propagation.

**Figure 2:**
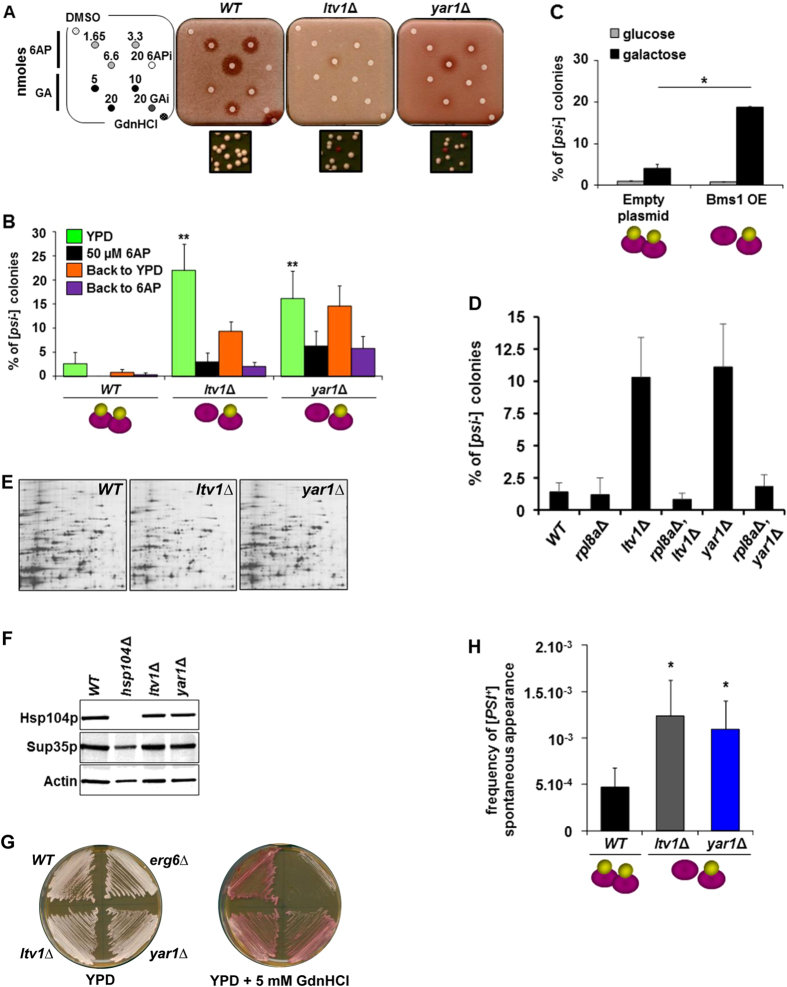
PFAR involvement in [*PSI*^+^] propagation and *de novo* appearance. (**A**) *WT, ltv1*Δ and *yar1*Δ [*PSI*^+^] strains were spread on GdnHCl-supplemented medium (200 μM). DMSO (compound vehicle) and GdnHCl (3 μmoles) were applied to top left and bottom right filters, respectively (see Supporting experimental procedures for further details). Bottom panels: *ltv1*Δ *and yar1*Δ colony phenotypes. **(B)** [*PSI*^+^] stability was evaluated by scoring [*psi*^−^] colonies appearing from [*PSI*^+^] cells as percentage of total cells for *WT* and *ltv1*Δ and *yar1*Δ strains. The effect of 6AP on [*PSI*^+^] stability was evaluated by scoring [*psi*^−^] red colonies from [*PSI*^+^] cells plated on medium supplemented with 50 μM 6AP. 6AP treatment reversibility was analyzed by spreading [*PSI*^+^] cells from 6AP-containing plates on YPD plates (back to YPD) or on 6AP-containing plates (50 μM) as control (back to 6AP). **(C)** [*PSI*^+^] *WT* weak strain was transformed either by a plasmid expressing *BMS1* under a galactose-induced promoter or by empty vector. [*PSI*^+^] stability was evaluated by scoring [*psi*^−^] colonies from [*PSI*^+^] cells on a medium containing glucose (gray bars) or galactose (black bars). **(D)** [*PSI*^+^] stability was evaluated by scoring [*psi*^−^] colonies appearing from [*PSI*^+^] cells as percentage of total cells for *WT, rpl8a*Δ, *ltv1*Δ, *rpl8a*Δ/*ltv1*Δ, *yar1*Δ and *rpl8a*Δ/*yar1*Δ strains. **(E)** Protein expression profiles of *ltv1*Δ and *yar1*Δ mutants were analyzed by 2D-gel electrophoresis. **(F)** Hsp104p and Sup35p expression was analyzed by SDS-PAGE. Actin was used as a loading control. **(G)** [*PSI*^+^] *WT*, *ltv1*Δ*, yar1*Δ and *erg6*Δ cells were streaked on plates containing 0 or 5 mM GdnHCl. **(H)** *De novo* [*PSI*^+^] colony formation from *ltv1Δ* and *yar1Δ* [*psi*^−^] cells was monitored on adenine-free medium where only [*PSI*^+^] cells could grow. [*PSI*^+^] status of white and pink colonies growing on adenine-free medium was confirmed by curing with GdnHCl. Averages of 4 experiments including 5 technical repeats are shown with error bars. Bar height of panels (B,C,H) represents the mean; *P < 0.05 or **P < 0.001 on *t*-test versus *WT* cells grown on YPD (panel B), cells transformed with empty plasmid and grown on galactose-containing medium (panel C), or *WT* strain (panel H).

PFAR-enriched strains were then used to determine the effect of PFAR enrichment on [*PSI*^+^] propagation. As PFAR is the cellular target of the two antiprion drugs 6AP and GA, we first evaluated their ability to cure [*PSI*^+^] on PFAR-enriched strains *ltv1*Δ and *yar1*Δ. [*PSI*^+^] cells form white colonies whereas [*psi*^−^] cells form red colonies. GdnHCl was added to the medium to partially inhibit Hsp104p and thus favor the activity of drugs acting on conserved prion-controlling pathways distinct from Hsp104p^10^. The appearance of a halo of red colonies around a filter identifies a compound potentially active against [*PSI*^+^] prion^28^. Red halos around filters spotted with 6AP and GA were much smaller for *ltv1*Δ and *yar1*Δ mutants than *WT* strain (Fig. 2A) that indicates less efficient [*PSI*^+^] curing (Figure S2A). The mutants had read-through levels (Figure S2B), membrane permeability (Figure S2C) and contained a prion strain similar to that of *WT* strain (Figure S2D), suggesting that 6AP and GA were actually titrated out by the excess of their PFAR target. We next investigated the impact of PFAR enrichment on [*PSI*^+^] propagation as the proportion of [*psi*^−^] red colonies from [*PSI*^+^] colonies during cell division. As shown in Fig. 2B, the proportion of [*psi*^−^] red colonies (YPD) was higher for *ltv1*Δ and *yar1*Δ mutants than *WT* strain: i.e., [*PSI*^+^] propagation was impaired in PFAR-enriched cells. Furthermore, the PFAR inhibitor 6AP restored [*PSI*^+^] stability in *ltv1*Δ and *yar1*Δ strains (Fig. 2B), which shows that PFAR enrichment itself, but not subunits imbalance, is responsible for impaired [*PSI*^+^] propagation. 6AP effect was reversible in cells grown on 6AP-free YPD medium (Fig. 2B, back to YPD) compared to 6AP-containing medium (Fig. 2B, back to 6AP). To confirm these data, we used Bms1p, a ribosome assembly GTPase involved in 40S subunit biogenesis, overexpression of which disturbs the 60S:40S ratio in favor of 60S subunits^29^ (Figure S2E). Like in *ltv1*Δ and *yar1*Δ strains, Bms1p overexpression in a *WT* [*PSI*^+^] strain made [*PSI*^+^] less susceptible to 6AP and GA antiprion drugs (Figure S2F) and reduced its propagation rate, as shown by the higher proportion of red [*psi*^−^] colonies compared to *WT* strain (Fig. 2C). This agrees with the finding that Bms1p overexpression increased the rate of natively folded proteins heterologously overexpressed in yeast^29^.

In order to check that [*PSI*^+^] instability was a direct consequence of PFAR enrichment, we first analyzed [*PSI*^+^] propagation in *rpl8a*∆ cells which contain an increased amount of free 40S subunits^30^ and have a reduced growth rate similar to that of *ltv1*∆ and *yar1*∆ strains: [*PSI*^+^] stability was not altered in *rpl8a*∆ cells (Fig. 2D). Moreover, reducing free cytoplasmic rate of both 40S and 60S subunits by deleting *LTV1* or *YAR1* together with *RPL8A* did not affect [*PSI*^+^] stability despite the reduced growth rate of the double mutants *ltv1*∆/*rpl8a*∆ and *yar1*∆/*rpl8a* (Fig. 2D). These data showed that neither the decreased growth rate of *ltv1*∆ and *yar1*∆ cells nor the perturbation of subunit ratio is responsible for [*PSI*^+^] instability. We next analyzed *ltv1*Δ and *yar1*Δ mutants protein expression profiles: they were similar to that of *WT* strain (Fig. 2E), suggesting that global protein synthesis, in particular Hsp104p level (Fig. 2F), was not impaired in PFAR-enriched cells. Finally, as modification of Hsp70 expression level also leads to [*PSI*^+^] instability^31^, we checked that GdnHCl sensitivity was similar in *WT* and *ltv1*Δ and *yar1*Δ cells (Fig. 2G), indicating that Hsp70 function is not deficient in PFAR-enriched cells. Only *erg6*Δ cells (previously described as hypersensitive to guanidine toxicity^31^) were not able to grow in presence of 5 mM GdnHCl. Taken together, these data suggest that PFAR is a cellular modulator of [*PSI*^+^] propagation.

The study revealed a peculiar finding concerning the yeast [URE3] prion: PFAR enrichment in *yar1*Δ [URE3] strain (NT64 strain) dramatically increased [URE3] stability (Figure S2G), whereas [*PSI*^+^] stability was reduced in PFAR-enriched *ltv1*Δ and *yar1*Δ strains (Fig. 2B). For unknown reasons this “*WT”* yeast strain has a low level of 60S subunits compared to the 74-D694 strain background (Figure S2H). Thus the deletion of *YAR1*, instead of massively enriching cells in 60S free subunits, only restored a PFAR level better sustaining [URE3] propagation. Thus, propagation of [URE3], like [*PSI*^+^], relies on PFAR. However, PFAR enrichment of the NT64 [URE3] strain only restored a PFAR level maintaining [URE3] propagation.

We next investigated whether PFAR is also involved in spontaneous generation of [*PSI*^+^] and observed that its rate was slightly but significantly higher in *ltv1*Δ and *yar1*Δ strains than *WT* (Fig. 2H). We concluded that enhancing PFAR potentiates Sup35p conversion to the prion state, suggesting that PFAR helps Sup35p reach a prion-inducing conformation. Together with the impact of PFAR enrichment on [*PSI*^+^] propagation, this highlighted the role of PFAR in prionization.

### Alteration of PFAR rRNA nucleotides impairs [*PSI* ^+^] propagation

**Figure 3:**
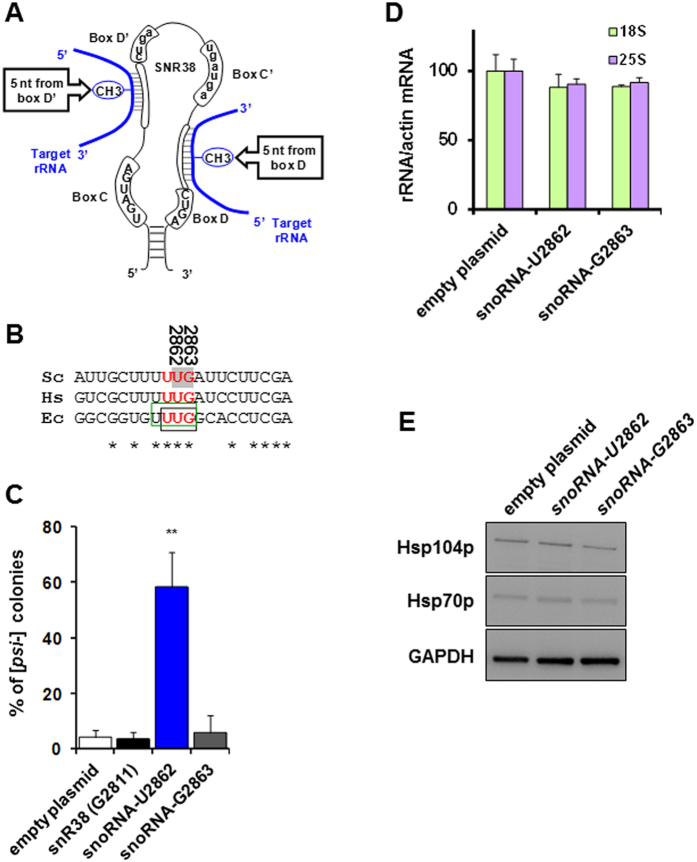
*In vitro* site-directed methylation of nucleotide U2862 belonging to PFAR active site impairs [*PSI*^+^] propagation. (**A**) Schematic structure of a canonical box C/D snoRNA. Members of the box C/D family contain a box C (UGAUGA) and box D (CUGA) positioned near the 5′ and 3′ termini respectively and brought closer by nearby short complementary sequences. Additional C- and D-like motifs (boxes C′ and D′) are present in the central region of many snoRNAs. Guiding 2′-*O*-methylation involves base pairing of the 10- to 21-nucleotide-long guide sequence positioned upstream of box D or D′ to the target rRNA, selecting the nucleotide positioned 5 bases upstream of D/D′ box for methylation (m). **(B)** Alignment of domain V rRNA sequences from *S. cerevisiae* (Sc, CP006467, bases 2803–2989), *Homo sapiens* (Hs, M11167.1) and *E. coli* (Ec, CP007136.1) surrounding U2862 (yeast numbering). The black box corresponds to *E. coli* domain V nucleotides involved in interaction with protein substrates^78^, and the green box to nucleotides interacting with 6AP and GA antiprion drugs^14^. *E. coli* domain V nucleotides showing loss of *in vitro* protein folding activity when mutated are shown in red^14^. Gray highlighting: nucleotides subjected to site-directed methylation. **(C)** *WT* [*PSI*^+^] cells were transformed by plasmids expressing PFAR-snoRNA specifically forcing methylation of U2862 and G2863 nucleotides involved in PFAR (gray, panel B). Negative controls: an empty plasmid (p424-*GPD*) and plasmid encoding natural snR38 snoRNA targeting G2811 and causing no phenotype. [*PSI*^+^] stability was assessed as the number of [*psi*^−^] red or sectored colonies from [*PSI*^+^] cells as a percentage of total cells. Averages of 3 experiments including 3 technical repeats are shown, with error bars; bar height represents mean; *t*-test: **P < 0.001 versus cells transformed by empty plasmid. **(D)** Relative abundance of amplified 18S and 25S rRNA was calculated using RT q-PCR, with *actin* mRNA as control for normalization in *WT* [*PSI*^+^] cells transformed with empty vector or plasmids encoding snoRNAs targeting U2862 and G2863 nucleotides. **(E)** Abundance of Hsp104p and Hsp70p, the expression of which was not modified in cells expressing PFAR-snoRNA U2862. GAPDH was used as loading control.

We next investigated the effect of PFAR inhibition on [*PSI*^+^] propagation by altering PFAR rRNA nucleotides using site-directed methylation^32^,^33^. Site-directed methylation is based on the hijacking of naturally encoded small nucleolar RNAs (snoRNA) to specifically methylate nucleotides at rRNA level and can be achieved by replacing the cellular snoRNA guide element by a custom guide sequence^32^. The nucleotide to be modified is targeted through specific base pairing of the snoRNA guide sequence with its rRNA substrate (Fig. 3A). Adding an unnatural methyl moiety to a rRNA specific nucleotide using the cellular machinery is equivalent to a gene-product point mutation^33^. We focused on U2862 and G2863 nucleotides which are collectively conserved in bacterial, yeast and human domain V (Fig. 3B), involved in ribosome protein folding *in vitro*^14^, able to bind 6AP and GA^14^ and not naturally methylated^34^. We thus generated PFAR-snoRNAs specifically targeting these two nucleotides of domain V of yeast 25S rRNA and expressed them in *WT* [*PSI*^+^] cells. [*PSI*^+^] stability was assessed as the proportion of [*psi*^−^] red colonies spontaneously appearing from *WT* [*PSI*^+^] white colonies expressing these PFAR-snoRNAs. Expression of PFAR-snoRNA targeting nucleotide U2862 led to a larger proportion of [*psi*^−^] red cells than cells transformed by PFAR-snoRNA targeting nucleotide G2863 and negative controls (Fig. 3C), suggesting nucleotide U2862 methylation markedly impairs [*PSI*^+^] propagation. As expected, forced methylation of U1757 known to induce growth defect in yeast^33^ and of catalytic adenine A2820, caused major growth interference and no transformants were obtained. [*PSI*^+^] instability in PFAR-snoRNA-U2862 expressing cells was not due to modification of the 25S:18S ratio (Fig. 3D)^35^ or to onset of cellular stress (induction of Hsp104p or Hsp70p expression, Fig. 3E). It is difficult to prove that the targeted nucleotide is methylated^32^,^35^, but it is highly likely that [*PSI*^+^] loss on expression of PFAR-snoRNA-U2862 was due to methylation rather than antisense interference^33^, as targeting methylation to the neighboring nucleotide G2863 caused no defect. These results thus clearly implicate PFAR in [*PSI*^+^] propagation with, to our knowledge, the first *in vivo* evidence of a direct link between inhibition of ribosome-assisted folding and prion destabilization.

### PFAR enrichment enables [*PSI* ^+^] propagation despite reduced Hsp104p activity

**Figure 4:**
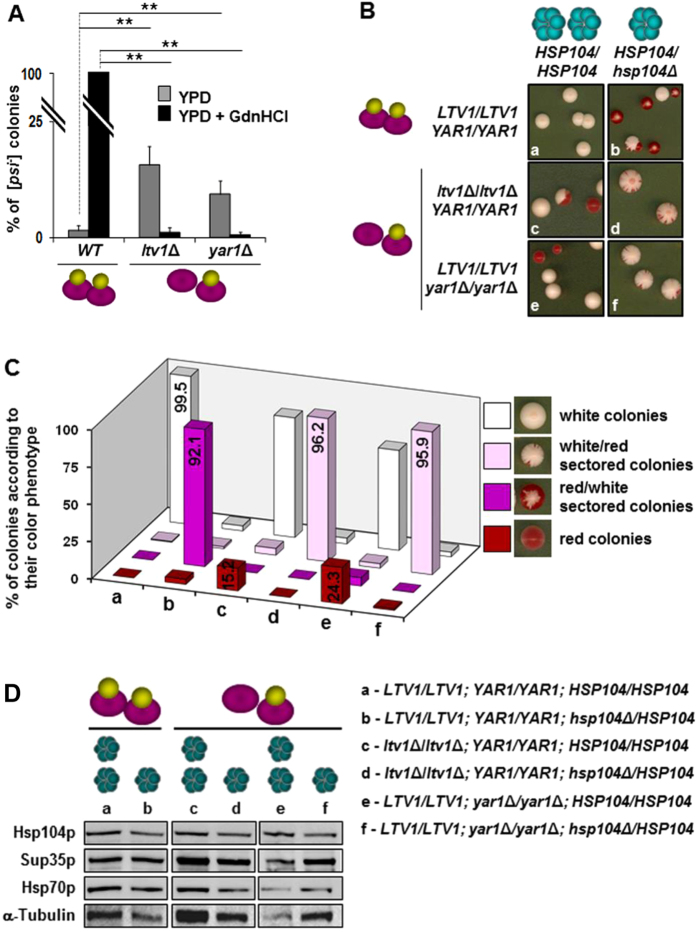
[*PSI*^+^] instability due to PFAR enrichment is counterbalanced by partial inhibition of Hsp104p activity. **(A)** [*PSI*^+^] stability in presence of GdnHCl was evaluated by scoring [*psi*^−^] colonies on YPD medium supplemented with 0.5 mM GdnHCl as a percentage of total cells for *WT, ltv1*Δ and *yar1*Δ strains. Experiments were repeated twice. A representative assay including 10 technical repeats is shown with error bars. Bar height represents the mean; *t-*test: **P < 0.001 versus untreated or GdnHCl-treated *WT* strain. (**B**) [*PSI*^+^] stability in *HSP104* haploinsufficient strains*. WT* diploid strains (*HSP104*/*HSP104*, *LTV1*/*LTV1*, *YAR1*/*YAR1,* panel a), *HSP104* haploinsufficient diploid strain (*HSP104*/*hsp104*Δ, *LTV1*/*LTV1*, *YAR1*/*YAR1,* panel b), PFAR-enriched diploid strains (*HSP104*/*HSP104, ltv1*Δ/*ltv1*Δ, panel c and *HSP104*/*HSP104, yar1*Δ/*yar1*Δ, panel e) and *HSP104* haploinsufficient, PFAR-enriched diploid strains *(HSP104*/*hsp104*Δ, *ltv1*Δ/*ltv1*Δ, panel d and *HSP104*/*hsp104*Δ*, yar1*Δ/*yar1*Δ, panel f) were spread on YPD medium to analyze [*PSI*^+^] phenotypes. The proportion of red sectors in a colony is proportional to [*PSI*^+^] instability. Plain red and sectored colonies were scored as [*psi*^−^]. According to observed phenotypes, sectored colonies were classified as white/red if they had a majority of white sectors, or red/white if a majority of red sectors. Red/white sectored colonies denote stronger [*PSI*^+^] instability than white/red. **(C)** Proportions of [*psi*^−^] red, [*psi*^−^] red/white sectored, [*psi*^−^] white/red sectored and [*PSI*^+^] white colonies produced by [*PSI*^+^] white colonies of the diploid strains described in the 6 panels of Fig. 4B, on YPD. Percentages are indicated in histogram bars. Experiments were repeated 3 times. A representative assay including 10 technical repeats is shown. **(D)** Lysates of exponentially growing diploid yeast cells were analyzed by SDS-PAGE for the expression of Hsp104p, Sup35p and Hsp70p. α-Tubulin was used as a loading control. The quantity of Sup35p and Hsp70p was not modified in *HSP104* haploinsufficient strains.

PFAR involvement in [*PSI*^+^] propagation resembles the role of Hsp104p in [*PSI*^+^] propagation, suggesting an interplay between Hsp104p and PFAR protein chaperone activities for [*PSI*^+^] propagation. This is supported by GdnHCl’s synergistic antiprion activity with 6AP and GA^10^,^13^. In the course of the present study, we also observed that the Hsp104p inhibitor GdnHCl was less effective in curing [*PSI*^+^] in *ltv1*Δ and* yar1*Δ mutants (Fig. 2A, bottom right filters) compared to *WT* strain (Figure S3A), despite similar GdnHCl sensitivity (Fig. 2G). Thus we reckoned that PFAR enrichment may partly compensate for Hsp104p’s inability to ensure [*PSI*^+^] propagation in presence of GdnHCl. However, despite PFAR enrichment, [*PSI*^+^] was lost in *ltv1*Δ*-hsp104*Δ and *yar1*Δ*-hsp104*Δ strains (data not shown): i.e., the PFAR level reached in *ltv1*Δ and* yar1*Δ strains was insufficient to fully compensate for *HSP104* deletion. We thus propose a model where Hsp104p and ribosome protein folding activities function complementarily to maintain stable [*PSI*^+^] propagation (Figure S3B, panels a,b). According to this model, the reduction in either of the two chaperon activities should be compensated by enrichment of the other (Figure S3B, panels c,d). With such a model, [*PSI*^+^] propagation should be sustained despite PFAR enrichment if Hsp104p activity is reduced (Figure S3B, panel c). To test this, we first evaluated [*PSI*^+^] behavior in PFAR-enriched mutants with Hsp104p inhibited by GdnHCl. Unlike the complete cure of *WT* strain [*PSI*^+^] prion in presence of GdnHCl, Hsp104p inhibition by GdnHCl increased [*PSI*^+^] stability 8 to 10-fold in *ltv1*Δ and *yar1*Δ strains compared to untreated [*PSI*^+^] cells (Figs 4A and S4A). Thus, [*PSI*^+^] instability caused by PFAR enhancement is compensated by reduced Hsp104p activity.

We then created diploid [*PSI*^+^] strains haploinsufficient for *HSP104* (i.e., in which one of the two *HSP104* copies was deleted, Fig. 4B): if PFAR enrichment compensates for partial loss of Hsp104p activity, [*PSI*^+^] should also be more stable in PFAR-enriched strains where one of the two *HSP104* alleles was deleted. As expected, similarly to haploid mutants, *ltv1*Δ/*ltv1*Δ and *yar1*Δ/*yar1*Δ diploid strains displayed a higher proportion of [*psi*^−^] red colonies than *WT* strain (Fig. 4B, panels a, c and e, and Figure S4A). The *HSP104*/*hsp104*Δ haploinsufficient [*PSI*^+^] strain produced many [*psi*^−^] red and red/white sectored colonies (92.1%, strain b Fig. 4B,C): i.e., *HSP104* haploinsufficiency in *WT* [*PSI*^+^] strain massively impaired prion propagation, without altering Sup35p protein levels (Fig. 4D). PFAR-enriched *ltv1*Δ/*ltv1*Δ and *yar1*Δ/*yar1*Δ *HSP104-*haploinsufficient strains produced many white/red sectored [*psi*^−^] colonies (~96% for *ltv1*Δ/*ltv1*Δ and *yar1*Δ/*yar1*Δ diploid strains, strains d and f on Fig. 4B,C): i.e., [*PSI*^+^] loss was lower in *HSP104-*haploinsufficient strains where PFAR is enriched (strains d and f) than *HSP104*/*HSP104* strains (strains c and e). Thus, PFAR enrichment sustained [*PSI*^+^] propagation when Hsp104p activity was at a level unable to efficiently maintain [*PSI*^+^] propagation (Figs 4B,C and S4A).

### Hsp104p overexpression enables [*PSI* ^+^] propagation when PFAR is reduced

**Figure 5:**
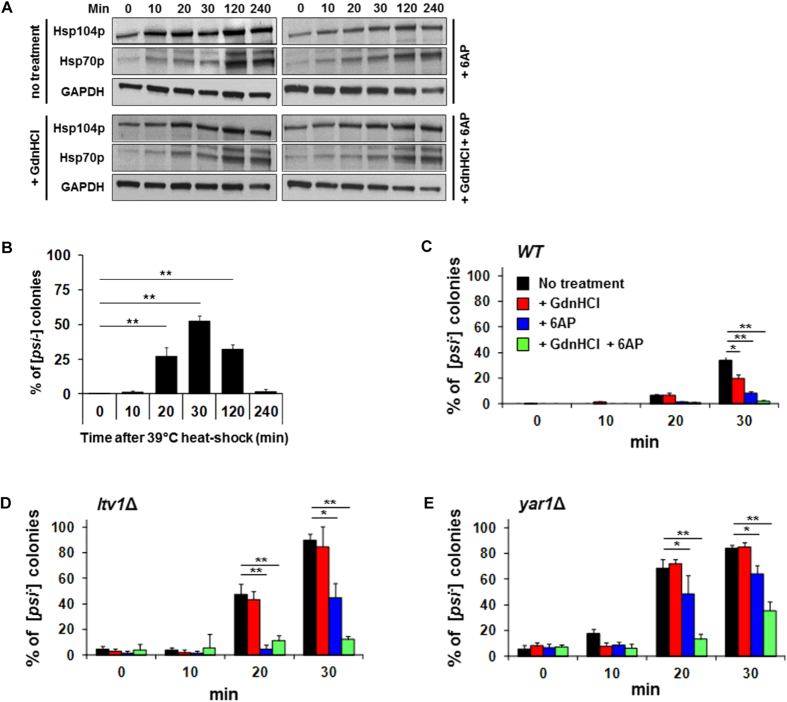
PFAR inhibition compensates [*PSI*^+^] loss in cells overexpressing Hsp104p. **(A)** *WT* [*PSI*^+^] cells were submitted to 39 °C moderate heat shock. *WT* cells harvested at indicated times were lysed and proteins were immunoblotted using anti-Hsp104p and anti-Hsp70p antibodies. Anti-GAPDH antibodies were used as a loading control. These data are representative of 3 different experiments. **(B)** *WT* cells harvested at indicated times were spread on YPD medium to score [*psi*^−^] colonies. Experiments were repeated 3 times. A representative assay including 6 technical repeats is shown with error bars. Bar height represents the mean; *t*-test: **P < 0.001. **(C–E)** [*PSI*^+^] stability increases in heat-shock cells treated by 6AP, GdnHCl or both. *WT* [*PSI*^+^] cells **(C)**, *ltv1*Δ [*PSI*^+^] cells **(D)** and *yar1*Δ [*PSI*^+^] cells **(E)** were pretreated by DMSO, 100 μM 6AP (*WT*) or 300 μM 6AP (*ltv1*Δ and *yar1*Δ), 1 mM GdnHCl or by 6AP + GdnHCl before 39 °C heat shock. Aliquots were collected at indicated incubation times, cells were spread on YPD plates and [*PSI*^+^] stability was evaluated by scoring [*psi*^−^] red or sectored colonies as percentage of total plated cells for untreated and 6AP-, GdnHCl-, 6AP- and GdnHCl-treated cells not heat-shocked. Experiments were repeated 3 times. A representative assay including 5 technical repeats is shown with error bars. Bar height represents the mean; *t*-test: *P < 0.05 or **P < 0.001 versus untreated strains.

As PFAR enrichment compensates for Hsp104p deficiency, we investigated whether enhanced Hsp104p activity could compensate for PFAR inhibition, as in our model (Figure S3B, panel d). It was recently shown that mild (39 °C) heat shock quickly induces Hsp104p overexpression and impairs [*PSI*^+^] segregation during cell division, due to imbalance between Hsp104p and other stress-inducible heat-shock proteins such as Hsp70p^36^. [*PSI*^+^] destabilization was maximal 30 min after heat shock when imbalance between Hsp104p and Hsps was at its peak^36^. Moderate heat shock induced early overexpression of Hsp104p and a delayed Hsp70 expression (Fig. 5A) and destabilized [*PSI*^+^] compared to non-shocked cells (Fig. 5B). In line with our hypothesis, [*PSI*^+^] stability in *WT* cells was less affected when cells were pre-treated with 6AP or both 6AP and GdnHCl (Figs 5C and S4B). [*PSI*^+^] was massively destabilized in heat-shocked PFAR-enriched cells (Figs 5D,E and S4B) and 6AP treatment restored [*PSI*^+^] stability in synergy with GdnHCl in *ltv1*Δ and *yar1*Δ PFAR-enriched strains (Figs 5D,E and S4B): i.e., PFAR and Hsp104p compensate each other for efficient [*PSI*^+^] propagation. Thus, a delicate balance between Hsp104p and PFAR chaperone activities appears necessary to maintain [*PSI*^+^] propagation (Figure S3B).

## Discussion

The present results are, to our knowledge, the first evidence of a direct link between PFAR, the protein folding activity of the ribosome, and [*PSI*^+^] propagation, and are a major breakthrough in understanding the conserved cellular mechanisms governing prion propagation and spontaneous appearance. Although the primary role of chaperones is to prevent protein misfolding and aggregation, involvement of cell chaperones like Hsp104p and PFAR in the propagation of a prion conformation is self-evident, as prion-forming proteins exist in several conformations and replication corresponds to propagation of differentially folded states^5^. Despite we cannot exclude that the action of PFAR may be indirect, [*PSI*^+^] loss was not a consequence of Hsp70 disturbance (Fig. 2G).

That [*PSI*^+^] is impaired when PFAR is inhibited or strengthened is reminiscent of Hsp104p’s involvement in prion propagation in yeast, just as [*PSI*^+^] propagation is impaired by both Hsp104p inhibition and overexpression. Like Hsp104p, domain V of the ribosome may have chaperone activity able to deal with cross-β sheet amyloid aggregates^37^ since reduced Hsp104p activity, deleterious for [*PSI*^+^] propagation, is compensated for by enriched PFAR activity, and *vice versa* (Figs 4 and 5). This interplay between PFAR and Hsp104p for [*PSI*^+^] propagation is in good agreement with GdnHCl’s synergistic effect with 6AP or GA in curing [*PSI*^+^] from yeast^10^,^13^.

That PFAR enrichment enhanced *de novo* [*PSI*^+^] formation (Fig. 2H) is similar to Hsp104p function, which greatly accelerates polymerization of the prion-forming domain (fragment NM) of Sup35 into amyloid fibers when added at sub-stoichiometric concentrations *in vitro*^38^. PFAR enrichment impairs [*PSI*^+^] propagation but boosts [*PSI*^+^] *de novo* formation, and indeed the two involve different mechanisms^39^. Neither GdnHCl nor excess Hsp104p, both known to impair [*PSI*^+^] propagation, prevents *de novo* appearance of [*PSI*^+^] by Sup35p overexpression in [*psi*^−^] strains. The fact that rRNA has previously been found associated to PrP^Sc^ fits nicely with our finding that PFAR is involved in *de novo* appearance of [*PSI*^+^]^40^. Thus, PFAR may well be involved in *de novo* formation of [*PSI*^+^], for which Hsp104p is not essential *in vivo*^39^,^41^.

Protein folding associated with an rRNA molecule furthers the list of ribozymes and non-coding RNAs serving a wide variety of biological functions^42^. There is now much evidence that PFAR may be an ancestral protein chaperone activity pre-dating the well-known protein-based chaperone Hsp104p disaggregase activity^17^. The “RNA world” hypothesis suggests that, prior to protein synthesis, catalytic functions were carried out by RNA molecules, with DNA and protein-based life developing later^43^. The universality of the antiprion effect of 6AP and GA, active both in yeast and mammals, is due to ribosome structure and function conservation throughout evolution, especially domain V of the large rRNA^44^. As there is no Hsp104p ortholog in mammals, and all three antiprion drugs 6AP, GA and IQ are active against both yeast and mammalian prions, it is tempting to speculate that PFAR is involved in prion formation and propagation in mammals. Of note, due to the localization of the ribosome, PFAR may only be involved in the propagation of cytosolic prions.

Although the actual cellular role of PFAR remains unclear, it may contribute to a wide variety of cell functions. Its potential *in vivo* importance in a variety of cell processes is highlighted by its chaperone activity on a wide range of protein substrates *in vitro*^17^. In previous reports, the bacterial ribosome showed general chaperone activity, acting as a holdase to prevent aggregation of denatured proteins and improve their folding^37^. Our basal thermotolerance and luciferase refolding assays in yeast clearly showed that, in absence of Hsp104p activity, PFAR efficiently participates in refolding proteins damaged by heat shock and in cell survival (Fig. 1B–E).

Thus, in addition to being directly involved in prion propagation, PFAR can also handle stress-induced protein aggregates and partly compensate for the absence of Hsp104p (Fig. 1). On the one hand, Hsp104p’s disaggregase activity confers a capacity to handle amyloid aggregates that is lacking in bacteria ClpB itself^4^. On the other hand, its capacity to deal with disordered aggregates is less than that of ClpB^4^; loss of this may have been viable as the ribosome possesses a compensatory activity of unknown nature. Together with Hsp104p and PFAR, yeast cells possess partly redundant disaggregase activities to efficiently handle stress-induced aggregates and tolerate amyloids. The absence of Hsp104-like disaggregase activity in multicellular organisms is likely due to stem-metazoan lineage gene loss preceding evolution of the gastrulation process^45^. This is particularly perplexing as the metazoan protein-based disaggregase system - Hsp110, Hsp70 and Hsp40 - cannot rapidly disaggregate amyloids^46^, even though some small heat-shock proteins can potentiate their amyloid-depolymerizing activity^47^. The recent identification of RuvbL1 and RuvbL2 disaggregases may shed a new light on this field^48^. As metazoa have no Hsp104p-like disaggregase activity, some groups explored reinforcing the metazoan cell proteostasis network with Hsp104p to help overcome the toxicity of amyloid-forming proteins. Hsp104p overexpression reduced aggregate formation in yeast^49^, *C. elegans*^50^, Drosophila^51^ and human cell lines^52^, and increased nerve-cell stress tolerance^53^, reduced polyglutamine aggregation and prolonged survival in transgenic mouse models of Huntington’s^54^ and Parkinson’s diseases^55^. Hsp104p may have evolved its operational plasticity in order to tolerate prions, possession of which confers selective advantage to cells in some conditions^4^,^56^. We can now extend this hypothesis: the ribosome may have retained RNA-based protein chaperone activity to compensate for the lack of protein-based Hsp104p-like disaggregase activity in bacteria and metazoan, and PFAR was maintained or evolved in yeast to combine with Hsp104p to take advantage of the numerous amyloid-prone proteins^57^. The protein folding activity of domain V of the ribosome might correspond to the Hsp104-like activity responsible for amyloid handling that is absent in metazoa. This hypothesis, while speculative, makes sense in light of recent findings that some antiprion compounds with anti-PFAR activity are also active in models of other protein aggregation-based diseases, where etiology is likely overlapping^58^,^59^: e.g., 6AP & GA interfered with aggregation cell-based models of Huntington’s disease (M. Blondel & A. Bertolotti, European patent application) and yeast-based models for Huntington’s and Parkinson’s disease (CV, JE, MB, unpublished data). Also, 6AP & GA showed benefit in a Drosophila-based model of OPMD (Oculopharyngeal muscular dystrophy), an inherited dominant myodegenerative disease caused by extension of a polyalanine tract in PABPN1 protein resulting in amyloid fiber accumulation^19^. Remarkably, synergy was observed between smaller deletions of rRNA encoding locus, which have no effect on OPMD phenotype, and sub-effective concentrations of 6AP & GA. Both increasing and decreasing the level of PFAR, a natural eukaryotic cell protein chaperone activity, may thus offer a new therapeutic avenue in some amyloid-based diseases.

## Methods

### Yeast strains and genetic manipulations

The yeast strain used in this study was 74-D694 *WT* [*PSI*^+^] weak (*Mata*, *ade1-14, trp1-289, his3Δ200, ura3-52, leu2-3,112*)^10^,^60^,^61^. Yeasts were grown and used as previously described^60^. As for PrP, various strains of yeast prions have also been described. They are based on the same prion protein but present different biological or biochemical properties due to structural differences^1^,^62^. Strong [*PSI*^+^] variant has a larger proportion of aggregated Sup35p than the weak variant, thus allowing a more efficient translational read-through than the weak variant, which is therefore pinker^1^. As the overall stability of strong [*PSI*^+^] variant is higher than the stability of the weak variant, our experiments were performed with weak [*PSI*^+^] variant, in which [*PSI*^+^] destabilization (appearance of [*psi*^−^] red colonies) was easier to detect.

Standard genetic manipulations were as described^63^. Mutant *ltv1*Δ, *yar1*Δ, *hsp104*Δ and *rpl8a*Δ strains were constructed by replacing, in 74-D694 weak [*PSI*^+^], *WT* genes by PCR-amplified of the heterologous *Escherichia coli kan*^*r*^ or *Schizosaccharomyces pombe his5*+ genes as selection markers^64^ using the primers described in Table S1, designed to replace the entire coding region of *LTV1*, *YAR1, HSP104* and *RPL8A* genes from ATG to stop codon. *ltv1*Δ strain (*ltv1*::*KanMx*) was constructed by replacing *LTV1* gene by *kan*^*r*^ marker using LTV1-F and LTV1-R primers. *yar1*Δ [*PSI*^+^] strain (*yar1*::*his5*) was constructed by replacing *YAR1* gene by *his5*+ marker using primers YAR1-F and YAR1-R. *hsp104*Δ-K and *hsp104*Δ-H strains were constructed by replacing *HSP104* gene by either *kan*^*r*^ (*hsp104*::*KanMx*) or *his5*+ (*hsp104*::*his5*) markers, respectively, using HSP104-F and HSP104-R primers. *rpl8a*Δ strain (*rpl8a*::*KanMx*) was constructed by replacing *RPL8A* gene by *kan*^*r*^ marker using RPL8A-F and RPL8A-R primers. After transformation of yeast cells with the deletion cassette by the lithium acetate procedure, a successful gene replacement was demonstrated by analytical PCR on genomic DNA using LTV1-Fbis and LTV1-Rbis, YAR1-Fbis and YAR1-Rbis, HSP104bis-F and HSP104bis-R, and RPL8Abis-F and RPL8Abis-R primers (Table S1).

*WT* diploid strain (*HSP104*/*HSP104*, *LTV1*/*LTV1*, *YAR1*/*YAR1*), *HSP104* haploinsufficient diploid strain (*HSP104*/*hsp104*Δ, *LTV1*/*LTV1*, *YAR1*/*YAR1),* PFAR enriched diploid strains (*HSP104*/*HSP104, ltv1*Δ/*ltv1*Δ and *HSP104*/*HSP104, yar1*Δ/*yar1*Δ) and PFAR enriched *HSP104* haploinsufficient diploid strains *(HSP104*/*hsp104*Δ *ltv1*Δ/*ltv1*Δ and *HSP104*/*hsp104*Δ*, yar1*Δ/*yar1*Δ) were obtained by mating *HSP104, hsp104*::*KanMx*, *hsp104*::*his5*, *ltv1*::*KanMx* and *yar1*::*his5* haploid strains described above. *ltv1*Δ/*rpl8a*Δ and *yar1*Δ/*rpl8a*Δ strains were obtained by mating *ltv1*::*KanMx, yar1*::*his5* and *rpl8a*::*KanMx* haploid strains described above.

Mutant *yar1*Δ NT64 [URE3] strain was constructed by replacing, in NT64 [URE3] (*MATα, trp1-1, ade2-1, his3-11,15, leu2-3,112, ura3-1, pdal5::ADE2*)^10^, *YAR1* gene by *his5*+ marker^64^ using primers YAR1-F and YAR1-R described in Table S1.

*BMS1*^65^ complete ORF (YPL217C) cloned in Gateway vector under *GAL1* promoter (Open Biosytems, plasmid 28A6) came from the Yeast ORF collection of E. Phizicky and M. Snyder^66^. Bms1p expression was induced on medium containing 2% galactose and 2% raffinose. pDCM90 plasmid (gift by D. Masison) allows constitutive expression of a temperature-sensitive luciferase polypeptide^67^.

### Basal thermotolerance

[*psi*^−^] *WT*, *ltv1*Δ, *yar1*Δ, *hsp104*Δ, *hsp104*Δ/*ltv1*Δ and *hsp104*Δ/*yar1*Δ 74-D694 strain cells were grown in liquid YPD medium at 29 °C and exponentially growing cells were diluted to 2,500 cells/ml. Cultures were split into 2 samples and moved to 29 °C (no heat shock) and to 47 °C (heat shock). Aliquots were taken after specified periods and cells were spread for viability onto YPD medium and incubated at 29 °C for 5 days^68^. Viability was monitored as the number of surviving heat shocked cells versus cells left at 29 °C for each strain.

### *In vivo* luciferase refolding assay

[*psi*^−^] *WT*, *ltv1*Δ, *yar1*Δ, *hsp104*Δ, *hsp104*Δ/*ltv1*Δ and *hsp104*Δ/*yar1*Δ 74-D694 strains were transformed with pDCM90 plasmid^69^,^70^, allowing constitutive expression of temperature-sensitive luciferase polypeptide. Heat-inactivated luciferase activity was monitored as described in ref. 71. Briefly, transformants were exponentially grown at 29 °C. Cells were pretreated for 30 min at 37 °C to induce expression of heat-shock proteins. Cells were treated with 200 μM 6AP or DMSO before heat-shock. Luciferase was then heat-inactivated by incubation at 45 °C for 60 min or kept at 29 °C for 60 min as control (pre-heat-shock). 10 μg/mL cycloheximide (Sigma Aldrich) was added after 50 min at 45 °C to arrest luciferase synthesis during recovery. Luciferase activity was immediately measured after 60 min of heat-shock at 45 °C (0 min) and cells were then left to recover at 25 °C for indicated periods, luciferase activity being assessed at 30 min intervals by adding 10 μl n-decylaldehyde (Sigma Aldrich) to 200 μL yeast culture. Luminescence was quantified using a Varioscan microplate reader (ThermoFisher). Luciferase activity was then expressed as a percentage of the activity before the 45 °C heat treatment for each strain.

### Polysome gradients

A total of 18 OD_600_ of exponentially growing yeast cells were treated for 10 min with 10 μg/ml of cycloheximide, harvested by centrifugation, and washed cell pellets were resuspended in 150 μl lysis buffer (10 mM Tris-HCl pH 7.5, 100 mM NaCl, 30 mM MgCl_2_, 200 μg/mL heparin, 10 μg/mL cycloheximide, 1 mM DTT, 40 U/mL RNase inhibitor, antiprotease cocktail (Roche), in DEPC-treated water). After the addition of 425–600 μm glass beads (Sigma Aldrich), cells were lysed by the alternating of 6 vortexing and ice-cooling steps of 30 sec each and then centrifuged for 5 min at 2,300 g at 4 °C. Supernatants were recovered, centrifuged at 9,300 g for 10 min at 4 °C and assayed for RNA content at OD_260_. 100 U OD_260_ of RNA were loaded on top of 10–40% sucrose gradients, centrifuged for 3h at 41,600 rpm using a SW55ti swing rotor (Beckman coulter). 200 μL fractions were collected, and OD_260_ was measured on 1/10 diluted fractions^26^,^72^.

### Pulse chase

5 ml of exponentially growing cultures of *WT*, *ltv1*Δ and *yar1*Δ [*PSI*^+^] cells were grown in presence of radiolabeled [^35^S] methionine (PerkinElmer Life Sciences) for 10 min, and then chased in medium containing 50 mM cold methionine for 15 min before harvesting. Protein extracts were prepared as described above and analyzed by 2D-gel electrophoresis followed by autoradiography.

### Electrophoresis, western blot and antibodies

A total of 6 OD_600_ of exponentially growing yeast cells were harvested by centrifugation and cell pellets were resuspended in lysis buffer (25 mM Tris-HCl pH 7.4, 100 mM NaCl, 0.2% Triton X-100, antiprotease cocktail (Roche), 1 mM phenyl-methylsulfonyl fluoride). After the addition of 425–600 μm acid-washed glass beads (Sigma Aldrich), cells were lysed by the alternating of six vortexing and ice-cooling steps for 30 sec each and then centrifuged for 3 min at 800 g at 4 °C. Supernatants were recovered and assayed for protein content. Protein extracts were analysed by 10% SDS-PAGE (precast NuPAGE, Invitrogen) and transferred to 0.45 μm nitrocellulose membranes (Whatman). Membranes were blocked with PBS 1X/0.1% Igepal containing 5% fat-free milk and 0.5% BSA, and incubated overnight at 4 °C using 1/1,000 rabbit anti-Sup35p serum (made by Eurogentec according to^73^), 1/10,000 mouse monoclonal anti-Hsp104p (kind gift of J. Glover, Montreal), 1/5,000 anti-HA (Clontech, cat. 631207), 1/5,000 mouse anti-Actin (Calbiochem, cat. CP01), 1/2,500 mouse anti-Hsp70/Hsc70 (Stressgen, cat. ADI-SPA-822-D), or 1/5,000 anti-GAPDH (Abcam, cat. ab125247). The membranes were then washed with fresh PBS 1X/0.1% Igepal and incubated for 45 min with secondary antibodies (goat anti-rabbit or goat anti-mouse, Biorad; goat anti-mouse IgM, Calbiochem) conjugated to horseradish peroxydase at a 1/3000 dilution, and analysed by Enhanced Chemiluminescence (ECL, Amersham) using Vilber-Lourmat Photodocumentation Chemistart 5000 imager.

### Yeast-based antiprion assay

An aliquot of exponentially growing cultures (170 μl of 0.5 OD_600_ culture of *WT* [*PSI*^+^] and 340 μL of 0.5 OD_600_ culture of *ltv1*Δ and *yar1*Δ [*PSI*^+^] strains) was homogeneously spread using sterile glass beads (≈3 to 5 mm diameter) on square (12 cm × 12 cm) Petri plates containing YPD solid medium supplemented with 200 μM GdnHCl, as previously described^10^,^60^. GdnHCl and guanabenz were purchased from Sigma Aldrich. Small sterile filters (Thermo-Fisher) were then placed on the agar surface and individual compounds were applied to each filter. DMSO, the vehicle, was applied to the top left filter as a negative control, and GdnHCl solubilized in water was applied to the bottom right filter as a positive control. The Petri plates were then incubated for four days at 25 °C and scanned using Snap Scan1212 (Agfa). In [*psi*^−^] cells, Sup35p is in a soluble form and causes the ribosome to stop translation at stop codons, including the premature stop codon present in the *ade1-14* allele of the *ADE1* gene. This leads to the production of a truncated and non-functional Ade1 protein, thus preventing cells to grow on medium lacking adenine and leading to the formation of red colonies on rich YPD medium because cells accumulate an intermediate metabolite of the adenine biosynthesis pathway (for review see^1^). When most of Sup35p is sequestrated under certain forms of amyloid, it cannot function efficiently in its normal role of translation termination, thus leading to an increased tendency of the ribosome to read through stop codons, including the premature stop codon in the *ade1-14* allele of *ADE1* gene^1^,^74^. Consequently, in cells harboring the *ade1-14* genotype, a functional Ade1p enzyme is produced in [*PSI*^+^] cells, allowing cells to form white or pink colonies on YPD rich medium. Thus, when a compound is active against [*PSI*^+^] prion, a halo of red colonies appears around the filter where it was spotted, whereas colonies remain white in the case of inactive compounds.

### Prion stability

50–100 cells from [*PSI*^+^] exponentially growing cultures were spread on ten YPD plates with specified drug concentrations and incubated at 29 °C. Following colony formation, [*PSI*^+^] loss was monitored as the percentage of red and sectored [*psi*^−^] colonies. Comparisons between strains and/or conditions were always performed within the same experiment.

### Frequency of spontaneous conversion from [*psi* ^−^] to [*PSI* ^+^]

The *de novo* formation of [*PSI*^+^] from [*psi*^−^] cells was monitored on media deprived of adenine where [*PSI*^+^] appearance was scored as growth, as previously described^41^,^75^,^76^. We used [*psi*^−^] [*PIN*^+^] cells for this set of experiment as [*PIN*^+^], the prion form of Rnq1 protein, is needed for [*PSI*^+^] *de no*vo formation^77^. 3 million cells from exponentially growing cultures (0.8 OD_600_ cultures) of *WT*, *ltv1*Δ and *yar1*Δ [*psi*^−^] [*PIN*^+^] strains were spread on synthetic medium lacking adenine (SD-Ade) and grown for 2 weeks at 29 °C. To distinguish [*PSI*^+^] colonies from chromosomal suppressor mutations, Ade^+^ colonies were subjected to treatment with 2 mM GdnHCl antiprion drug as genuine white/pink [*PSI*^+^] cells switch to [*psi*^−^] red cells when [*PSI*^+^] is cured by GdnHCl, whereas Ade+ spontaneous mutants keep a white color in presence of GdnHCl^41^. The number of colonies that underwent a colour change was scored for each replica plate. The total number of cells really spread was determined by plating, onto rich YPD medium, 100 μL of a 1/40,000 dilution of the same [*psi*^−^] [*PIN*^+^] cultures that were spread on SD-Ade.

### snoRNA site-directed methylation of rRNA

Some specific nucleotides of domain V of bacteria 23S rRNA were recently shown to specifically interact with denatured proteins under the refolding process^78^. Some of these nucleotides were also shown to interact with 6AP and GA, two drugs that display anti-PFAR and anti-prion activities^79^. The mutation of some of these nucleotides of bacterial 23S rRNA led to a complete loss of the refolding activity of domain V and completely abolished the interaction of 6AP and GA with rRNA at these positions^79^. The mutation of the corresponding nucleotides on domain V of yeast 25S rRNA also abolished its protein folding activity *in vitro*^79^. snoRNAs we used correspond to RNA moiety of snoRNA:protein complexes, named box C/D snoRNPs (small nucleolar RiboNucleoParticles), that are involved in 2′-O-methylation required for post-transcriptional maturation of rRNAs and other RNA produced in the nucleolus^80^,^81^. The specific guide sequence of snR38 (plasmid pBL150^33^) was replaced by a 14 nucleotides sequence targeting one of the nucleotides of 25S domain V we identified as involved in PFAR (see above)^79^. Primers were designed to create snoRNAs specifically targeting nucleotides U2862 and G2863 of domain V of rRNA 25S (PFAR-snoRNAs; Table S1). PFAR-snoRNAs were amplified from plasmid pBL143^33^ using each specific primer and BLO-38 primer (Table S1). PFAR-snoRNAs PCR products were digested by BglII and NdeI and cloned into pBL150^33^. PFAR-snoRNAs were then subcloned into p424 plasmid using BamHI and SacI restriction enzymes under the control of the strong constitutive *GPD* promoter. *WT* weak [*PSI*^+^] yeast cells were transformed by either p424-*GPD* encoding PFAR-snoRNAs, empty p424-*GPD* plasmid, or pBL150 plasmid encoding natural yeast snoRNA snR38 targeting G2811 as negative controls^33^. [*PSI*^+^] stability was then scored on YPD. snoRNAs targeting the catalytic adenine A2820 and U1757 on 18S rRNA (plasmid pBL143 encoding a snoRNA that has been shown to target a nucleotide essential for protein synthesis^33^), were used as positive controls inducing cell death (data not shown). Note that neither U2862 nor G2863 nucleotides of yeast 25S rRNA targeted by our custom PFAR-snoRNAs carry a natural post-translational modification^34^.

### RT Q-PCR

Total yeast cellular RNAs were extracted by RNAeasy and RN*ase*-free DN*ase* kits (QIAGEN). cDNA was synthesized from 1 μg DNA-free RNA by Superscript II (Invitrogen) using 18S-F, 25S-F and Actin-F primers (Table S1). cDNA samples underwent qPCR with 18S-F/18S-R, 25S-F/25S-R and Actin-F/Actin-R primers (Table S1), using the QuantiTect SYBR Green PCR kit (Qiagen) in Abiprism 7000 Sequence Detection System (Applied).

### Prion stability after moderate heat shock

*WT*, *ltv1*Δ and *yar1*Δ [*PSI*^+^] cells were grown in liquid YPD medium overnight at 29 °C and diluted to OD_600 _= 0.1, followed by incubation at 29 °C in presence of 6AP (1h at 300 μM for *ltv1*Δ and *yar1*Δ strains or 100 μM for *WT* strain), GdnHCl (15 min at 1 mM) or both 6AP and GdnHCl. Cultures were then shifted to 39 °C. Aliquots were taken after specified periods. Serial dilutions were prepared to plate 50 to 100 cells onto YPD solid medium and incubated at 29 °C^36^.

## Additional Information

**How to cite this article**: Blondel, M. *et al*. Protein Folding Activity of the Ribosome is involved in Yeast Prion Propagation. *Sci. Rep.* **6**, 32117; doi: 10.1038/srep32117 (2016).

## Supplementary Material

Supplementary Information
