# Supplementary material for: Protein Folding Activity of the Ribosome is involved in Yeast Prion Propagation
Source: Sci Rep. 2016 Sep 16;6:32117. doi: 10.1038/srep32117 (PMC5025663; doi:10.1038/srep32117)
Supplement: Supplementary Information [file srep32117-s1.pdf]

Protein Folding Activity of the Ribosome is involved in Yeast Prion Propagation

Marc Blondel[1], Flavie Soubigou[1§], Justine Evrard[1§], Phu hai Nguyen[1,3], Naushaba Hasin[2,4], Stéphane Chédin[5], Reynald Gillet[6], Marie-Astrid Contesse[1], Gaëlle Friocourt[1], Guillaume Stahl[7], Gary W. Jones[2,8], Cécile Voisset[1*].

Supplementary information

Supplementary Figures Legends

Figure S1. (A) Diagram of 40S:60S ratio modification in *ltv1*Δ or *yar1*Δ mutants (right) compared to *WT* (left). *ltv1Δ* and *yar1Δ* mutants contain more free 60S subunits in the cytoplasm than *WT* strain due to defective cytoplasm translocation of 40S subunits. **(B)** Survival of *WT*, *ltv1*Δ*, yar1*Δ*, hsp104*Δ*, ltv1*Δ*/hsp104*Δ and *yar1*Δ*/hsp104*Δ [*psi*-] cells used in **Fig. 1D and 1E** after specified post-heat-shock incubation periods.

Figure S2. (A) Cells from red halos surrounding filters on which 6AP or GA was loaded (**Fig. 2A**) were streaked on drug-free YPD medium. Cells surrounding filters on which DMSO and GdnHCl were loaded were used as negative and positive controls, respectively. **(B)** Read-through levels in *ltv1*Δ and *yar1*Δ [*psi*-] strains compared to *WT* [*psi*-] strain [1]. **(C)** The permeability of [*PSI*+] *WT, ltv1*Δ *and yar1*Δ strains was analyzed. Cells were spread on YPD medium and small filters were placed on the agar surface. 10 nmoles (filters 2, 3, 4) and 20 nmoles (filters 1, 5, 6, 7) of ethanol (filter 1), menadione (filters 2 and 5), Ifenprodil (filters 3 and 6) and chlorhexidine (filters 4 and 7), that are toxic for yeast cells, were applied to each filter. The non-growing halos caused by the four toxic compounds spotted on *WT*, *ltv1*Δ and *yar1*Δ strains had similar diameters, indicating that the reduced sensitivity to 6AP, GA and GdnHCl of [*PSI*+] prion in *ltv1*Δ and *yar1*Δ mutants was not due to a global change in membrane permeability. **(D)** [*PSI*+] *ltv1*Δ and *yar1*Δ strains were crossed with a *WT* [*psi*-] strain and the four cells issued from the diploid sporulation were tested for their sensitivity to 6AP and 6APi. The transmission of prion was faithful from *ltv1*Δ and *yar1*Δ [*PSI*+] cells to [*psi*-] *WT* cells, indicating that the resistance to prion curing in *ltv1*Δ and *yar1*Δ was not due to modification of the prion strain. **(E)** Polysome profiles of *WT* [*PSI*+] strain transformed by an empty plasmid (left panel) or by a plasmid allowing Bms1p overexpression (OE, right panel). Cells overexpressing Bms1p display an impoverishment in 40S subunits as well as an elevated level of free 60S subunits compared to the strain transformed by the empty plasmid. 60S:40S ratio was 1.48 for *WT* strain transformed by the empty vector p416-GAL1, and 2.69 for *WT* strain overexpressing Bms1p. The y axis shows arbitrary units. **(F)** *WT* [*PSI*+] cells transformed by an empty plasmid (left panels) or by the plasmid allowing Bms1p overexpression (right panels) were spread on glucose- (- panels) or 2% galactose-containing medium (+ panels) supplemented with 200 µM GdnHCl. Small filters were then placed on the agar surface and various

amounts of 6AP and GA were applied to each filter, except for the top left filter where DMSO was added (negative control). The size of the red halos is proportional to the efficiency of [*PSI*$^+$] curing. **(G)** [URE3] stability was evaluated by scoring [ure3-0] colonies appearing from [URE3] cells, as a percentage of total cells for *WT and yar1*Δ strains. Bar height represents the mean; *t*-test: $**P<0.001$ versus to *WT* cells grown on YPD. **(H)** Polysome profiles of *WT* and *yar1*Δ [URE3] strains showing that *yar1*Δ mutant strain displayed elevated levels of free 60S subunits, as well as an impoverishment in 40S subunits compared to *WT* strain that is particularly poor in 60S subunits.

Figure S3. (A) [*PSI*$^+$] is less efficiently cured by GdnHCl in PFAR-enriched strains than in *WT* strain. [*PSI*$^+$] *WT, ltv1*Δ *and yar1*Δ strains were spread on YPD medium. Small filters were then placed on the agar surface and various amounts of GdnHCl were applied to each filter, except for the top left filter where DMSO was added (negative control). The size of the red halos is proportional to the efficiency of [*PSI*$^+$] curing. **(B)** Model of the interplay between protein folding activities of Hsp104p and ribosome in modulating [*PSI*$^+$] propagation. We propose a model of how Hsp104p and PFAR may sustain together the propagation of [*PSI*$^+$] (**panel a**). **Panel b -** [*PSI*$^+$] propagation is similarly affected by enrichment in Hsp104p by heat shock or overexpression (upper left, [a2,3]) or PFAR by 60S enrichment (upper right, [b](this paper, **Fig. 2**)) or by inhibition of Hsp104p by GdnHCl (lower left, [c4]) or PFAR by 6AP (lower right, [d5]). **Panels c & d -** On this model for Hsp104p and PFAR interplay, reducing the activity of one could be compensated by enrichment of the activity of the other.

Figure S4. Summary of data presented in **Figure 4 (A)** and in **Figure 5 (B)** in the light of the model shown in **Figure S3B**.

Supplementary Table

Table S1 – primers used to create mutant strains and snoRNAs

Primer name	Primer sequence (5' to 3')
**Deletion primers**	
LTV1-F	GTATTT CAAAGA CTTTAA GGGGAA TATAAA AAGCAC GAAGCG GATCCC CGGGTT AATTAA
LTV1-R	GTACTT GTAATG TAGGTG CTTTCT CATCTC ATTCTA CTCCTG AATTCG AGCTCG TTTAA
YAR1-F	TATTTC CAAATA GAAAAA AAAAAT CTACAT ATACGC AGATCG GATCCC CGGGTT AATTAA
YAR1-R	ATTACG GCTTTT ATTCCA CGAAGA AAACAA GCTCTT TACTGA ATTCGA GCTCGT TTAAAC
HSP104-F	AAAGAA ATCAAC TACACG TACCAT AAAATA TACAGA ATATCG GATCCC CGGGTT AATTAA
HSP104-R	ATTCTT GTTCGA AAGTTT TTAAAA ATCACA CTATAT TAAAGA ATTCGA GCTCGT TTAAAC
RPL8A-F	CATTGCTTACCCTCTATTATCACATCAAAACAACTAATTCGAACGGATCCCCGGGTTAATTAA
RPL8A-R	ATTAAAAAATAAATTTTTTATGCAAAATTTCTCATTTTCAATGAATTCGAGCTCGTTTAAAC
LTV1-Fbis	GAATAT TATGAG CATCTA AATC
LTV1-Rbis	GCATCA ATGCAT TCTAGG C
YAR1-Fbis	GTGGTA ATATCA CCATGA ACG
YAR1-Rbis	GCATTT CTGCTG GTTCCA TC
HSP104bis-F	GAACTG CAAATT ATATCA CAG
HSP104bis-R	ATTATT CACAG CAAGAT GAAC
RPL8Abis-F	ATTACTATTCCAGTTGTCAG
RPL8Abis-R	CTTAAAAGGTTATTTAAGGTC
**snoRNA guide sequences and analysis primer**	
BLO-38	ATACATTAATAGATCTCCAAGAAACTACGC
snoRNA-U2954	GGGT**T**TAGACCGTC
snoRNA-U2862	TTTT**T**GATTCTTCG
snoRNA-G2863	TTTT**G**ATTCTTCGA
snoRNA-U2873	TCGA**T**GTCGGGCTCT
snoRNA-C2876	ATGT**C**GGCTCTTCC
snoRNA-U2932	CCAC**T**AATAGGGAA
snoRNA-G2957	TTTA**G**ACCGTCGTG
**RT Q-PCR primers**	
25S-F	AGACCGTCGCTTGCTACAAT
25S-R	ATGACGAGGCATTTGGCTAC
18S-F	TTGTGCTGGCGATGGTTCA
18S-R	TGCTGCCTTCCTTGGATGTG
Actin-F	ATGGTCGGTATGGGTCAAAAA
Actin-R	TTCCATATCGTCCCAGTTGGT

Supplementary References

1 Stahl, G., Bidou, L., Rousset, J. P. & Cassan, M. Versatile vectors to study recoding: conservation of rules between yeast and mammalian cells. *Nucleic acids research* **23**, 1557-1560 (1995).

2 Helsen, C. W. & Glover, J. R. Insight into molecular basis of curing of [PSI+] prion by overexpression of 104-kDa heat shock protein (Hsp104). *J Biol Chem* **287**, 542-556, doi:10.1074/jbc.M111.302869 (2012).

3 Wickner, R. B. *et al.* Amyloids and yeast prion biology. *Biochemistry* **52**, 1514-1527, doi:10.1021/bi301686a (2013).

4 Liebman, S. W. & Chernoff, Y. O. Prions in yeast. *Genetics* **191**, 1041-1072, doi:10.1534/genetics.111.137760 (2012).

5 Tribouillard-Tanvier, D. *et al.* Protein folding activity of ribosomal RNA is a selective target of two unrelated antiprion drugs. *PLoS ONE* **3**, e2174 (2008).

Figure S1

A

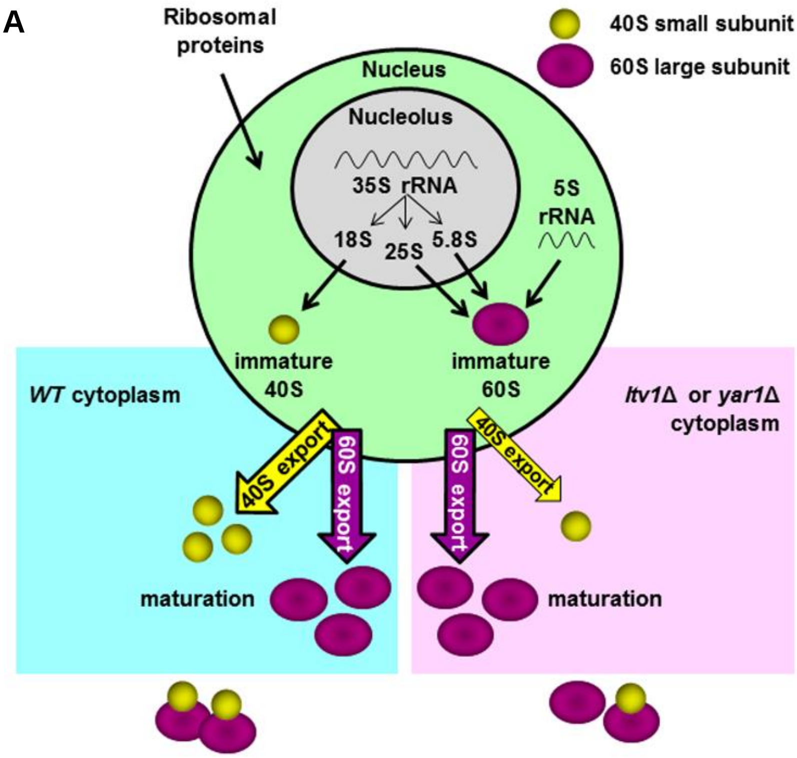
Ribosomal proteins
40S small subunit
60S large subunit
Nucleus
Nucleolus
35S rRNA
18S
25S
5.8S
5S rRNA
immature 40S
immature 60S
WT cytoplasm
ltv1Δ or yar1Δ cytoplasm
40S export
60S export
60S export
40S export
maturation
maturation

B

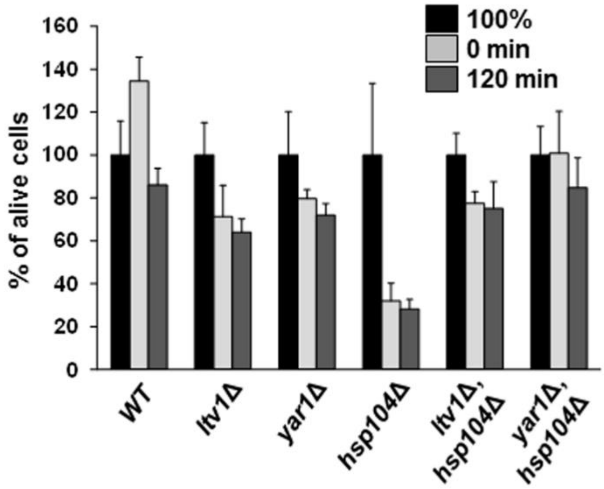
100%
0 min
120 min
% of alive cells
160
140
120
100
80
60
40
20
0
WT
ltv1Δ
yar1Δ
hsp104Δ
ltv1Δ, hsp104Δ
yar1Δ, hsp104Δ

Figure S2

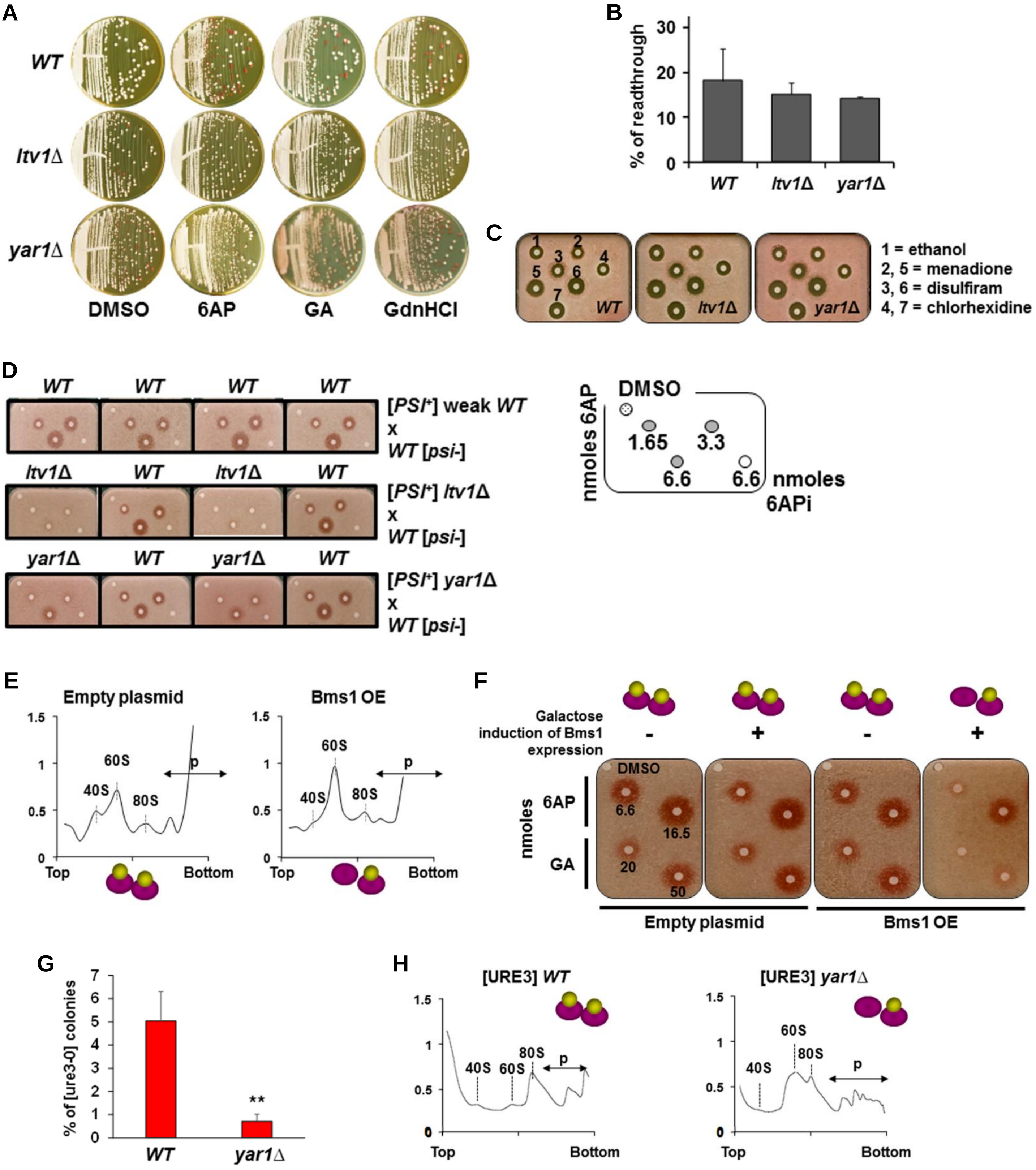

A
WT
ltv1Δ
yar1Δ
DMSO
6AP
GA
GdnHCl
B
% of readthrough
30
20
10
0
WT
ltv1Δ
yar1Δ
C
1 = ethanol
2, 5 = menadione
3, 6 = disulfiram
4, 7 = chlorhexidine
D
[PSI+] weak WT x WT [psi-]
[PSI+] ltv1Δ x WT [psi-]
[PSI+] yar1Δ x WT [psi-]
DMSO
nmoles 6AP
1.65
3.3
6.6
6.6
nmoles 6APi
E
Empty plasmid
Bms1 OE
40S
60S
80S
p
Top
Bottom
F
Galactose induction of Bms1 expression
-
+
nmoles
6AP
GA
6.6
16.5
20
50
Empty plasmid
Bms1 OE
G
% of [ure3-0] colonies
**
WT
yar1Δ
H
[URE3] WT
[URE3] yar1Δ
40S
60S
80S
p
Top
Bottom

Figure S3

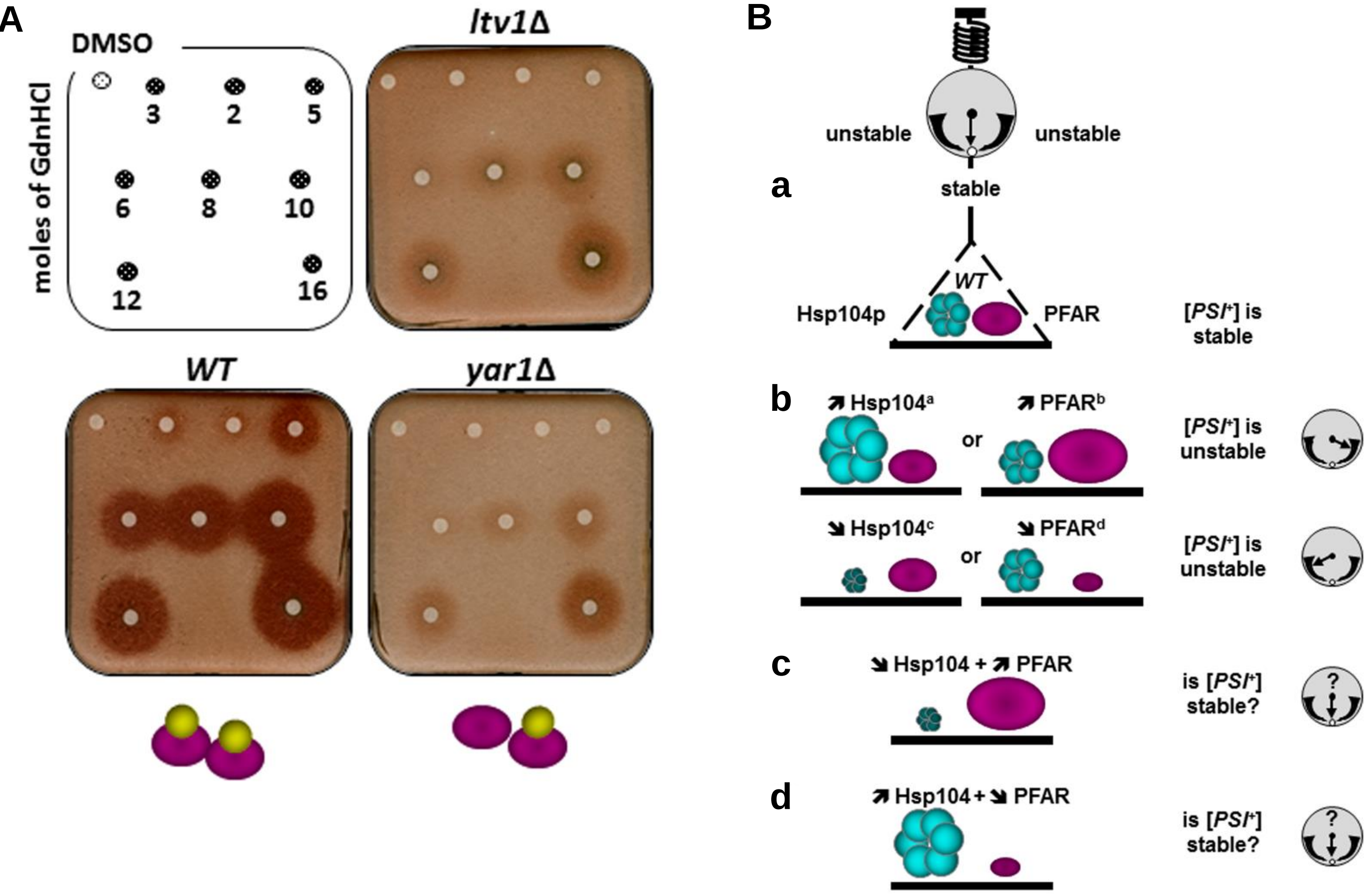
A
DMSO
moles of GdnHCl
3
2
5
6
8
10
12
16
ltv1Δ
WT
yar1Δ
B
unstable
unstable
a
stable
WT
Hsp104p
PFAR
[PSI+] is stable
b
Hsp104a
or
PFARb
[PSI+] is unstable
Hsp104c
or
PFARd
[PSI+] is unstable
c
Hsp104 + PFAR
is [PSI+] stable?
?
d
Hsp104 + PFAR
is [PSI+] stable?
?

Figure S4

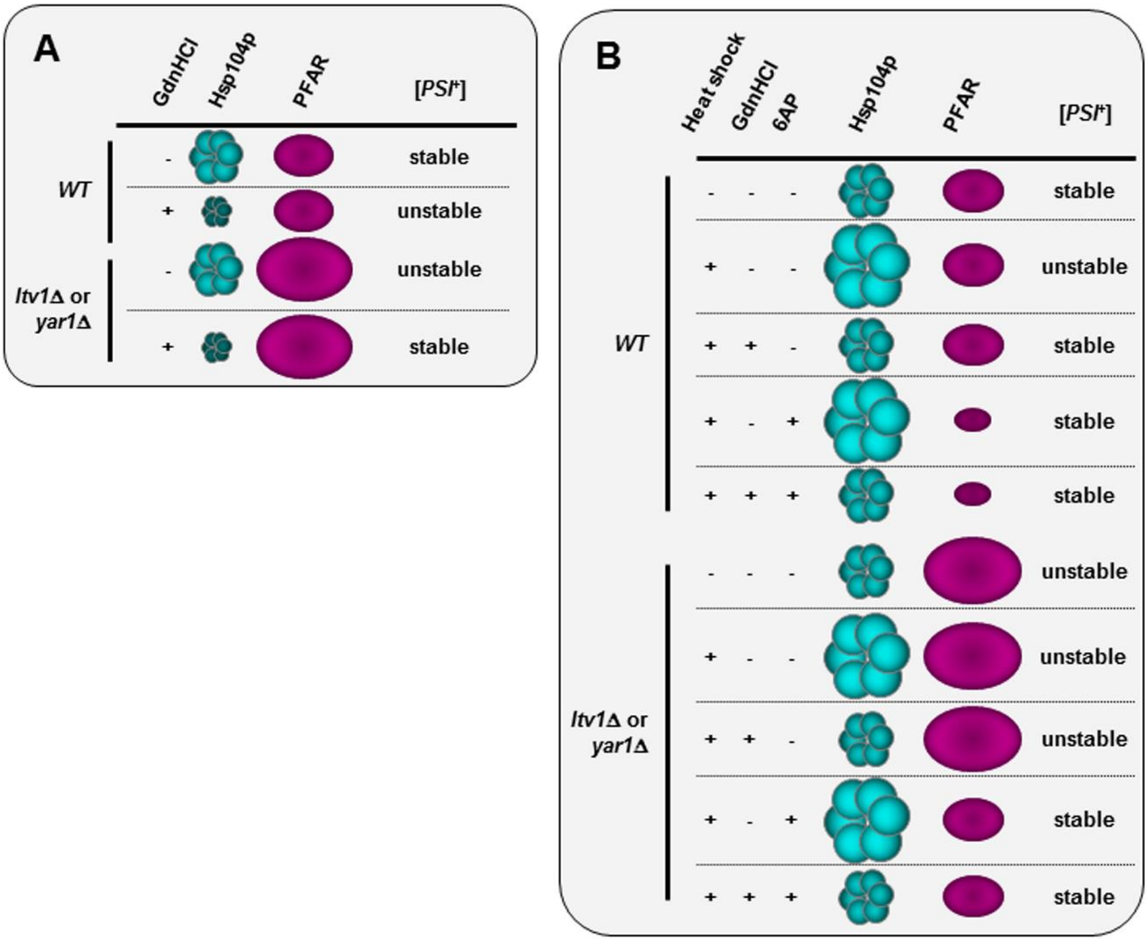
A
GdnHCl
Hsp104p
PFAR
[PSI+]
WT
ltv1Δ or yar1Δ
stable
unstable
unstable
stable
B
Heat shock
GdnHCl
6AP
Hsp104p
PFAR
[PSI+]
WT
ltv1Δ or yar1Δ
stable
unstable
stable
stable
stable
unstable
unstable
unstable
stable
stable
